# Selective vulnerability of AGTR1+ dopaminergic neurons in Parkinson's Disease lies at an intersection with Type 2 diabetes and hypertension

**DOI:** 10.1002/alz70855_102843

**Published:** 2025-12-23

**Authors:** Viola Volpato

**Affiliations:** ^1^ UKDRI Cardiff, Cardiff, cardiff, United Kingdom

## Abstract

**Background:**

By studying dysfunctional nigral cellular interactions and by identifying metabolic processes and comorbidities associated with AGTR1+ DaNs selective degeneration, we aim to analyse the molecular mechanisms for the loss of dopaminergic neurons (DaNs) in PD.

**Method:**

We generated the first deep and full length single nuclei transcriptomic atlas of the human substantia nigra, sequencing 23,885 nuclei from the postmortem ventral nigra of 7 healthy, 9 Parkinson's Disease (PD) and 4 Incidental Lewy Body Disorder patients.

**Result:**

Across cell types, the pattern of differential splicing was distinct to that of differential gene expression. PD genetic risk converged through splicing and expression onto dysfunctional cellular interactions affecting neuronal protection/support of AGTR1+ DaNs between perineuronal satellite oligodendrocytes, oligodendrocyte precursor cells and reactive astrocytes. Activation of the renin‐angiotensin system in AGTR1+ DaNs correlated with upregulation of synaptic transmission, dopamine transport but also with stress‐activated MAPK cascade and immune response. AGTR1+ DaNs genes also showed an enrichment in Type 2 diabetes (T2D) genetic risk and concomitantly the strongest upregulation of the T2D therapeutic target, GLP1R, whose agonists are being trialled as PD treatments. Notably, when performing a genome‐wide association analysis between PD patients diagnosed with T2D and those with no T2D history we found genome‐wide significant hits in both AGTR1 and the transcription factor TCF7L2 which is associated with neuronal survival and the regulation of GLP1R ligand expression. Furthermore, AGTR1+ DaNs, perineuronal satellite ODCs and reactive astrocytes also showed enrichment in hypertension genetic risk. The shared PD/hypertension risk genes may explain up to 25% of PD GWA loci effects and promote effective repurposing in PD of hypertension‐related drugs such as angiotensin blockers.

**Conclusion:**

Overall, our results provide genetically‐underpinned molecular mechanism through which promising treatments for PD may work.

